# Effect of Upright Posture on Endothelial Function in Women and Men

**DOI:** 10.3389/fphys.2022.846229

**Published:** 2022-03-24

**Authors:** Karim Habib, Behzad Fallah, Heather Edgell

**Affiliations:** ^1^School of Kinesiology and Health Science, York University, Toronto, ON, Canada; ^2^Muscle Health Research Center, York University, Toronto, ON, Canada

**Keywords:** flow-mediated dilation, reactive hyperemia, orthostatic stress, sex differences, hemodynamics

## Abstract

Women are more prone to orthostatic intolerance compared to men and have a greater vasodilatory capacity. We investigated the hypothesis that women would have greater peripheral flow-mediated dilation (FMD) while in the upright posture compared to men, which could contribute to this phenomenon. In young healthy women (age: 20 ± 3, BMI: 27 ± 5 kg/m^2^, *n* = 10) and men (age = 21 ± 2, BMI: 27 ± 8 kg/m^2^, *n* = 8), we assessed FMD of the brachial artery and hemodynamics to determine endothelial function during the supine and 70° head-up tilt postures (randomized). The brachial artery was kept at heart level in both trials. We observed that FMD increased in both sexes during tilt (Women: 11.9 ± 5.3 to 15.7 ± 5.6%; Men: 8.4 ± 3.2 to 14.6 ± 3.4%, Main effect of tilt *p* = 0.005) which was not due to changes in blood pressure or shear stress. There were no interaction effects between sex and posture. In a second cohort of women (age: 22 ± 3, BMI: 23 ± 3 kg/m^2^, *n* = 9) and men (age: 22 ± 2, BMI: 25 ± 8 kg/m^2^, *n* = 8), we investigated reactive hyperemia by peripheral arterial tonometry (LnRHI) *via* EndoPAT. Interestingly, we found that the EndoPAT response was decreased in both sexes during tilt (LnRHI: Men: 0.70 ± 0.28 to 0.59 ± 0.40, Women: 0.52 ± 0.23 to 0.30 ± 0.32, Main effect of tilt *p* = 0.037). We previously found that FMD is related to coronary responses to acetylcholine and adenosine whereas EndoPAT is related to coronary responses to dobutamine. Therefore, we suggest that sympathetic mediated dilation is attenuated in the upright posture while the increased vasodilatory response as measured by FMD in the tilt posture could be attributed to increasing metabolite production from postural muscles.

## Introduction

Orthostatic tolerance is well-known to be lower in women compared to men, yet the mechanisms are still being investigated. Previous studies have focused on hemodynamics and vasoconstrictor capacity, yet few have investigated vasodilatory capacity. Vasodilatory capacity could be an important consideration in orthostatic tolerance studies due to potential functional sympatholysis while upright. Greater vasodilation or sympatholysis while upright would reduce peripheral resistance and thus blood pressure, potentially contributing to orthostatic intolerance. A standard non-invasive measurement to assess vasodilatory capacity is brachial artery flow-mediated dilation (FMD). FMD has been shown to be enhanced in women compared to men (Hashimoto et al., 1995; Harris et al., 2012), and estradiol has been shown to upregulate the production of endothelial nitric oxide synthase (Rosenfeld et al., 2003; Kan et al., 2008). However, the sex differences in FMD have recently been disputed based on controlling for women having a smaller baseline diameter (i.e., allometric scaling, Shenouda et al., 2018; Johns et al., 2020). It has also been observed that women have greater activity/sensitivity of β2-adrenergic receptors, which cause peripheral vasodilation (Kneale et al., 2000). Since Fu et al. have observed similar sympathetic nerve responses during upright tilt between the sexes (Fu et al., 2005, 2010); the greater β2-adrenergic activity/sensitivity in women may result in greater adrenergic induced vasodilation.

Thijssen et al. (2014) observed that FMD was attenuated following simulated orthostatic stress *via* lower body negative pressure (LBNP), and Dyson et al. (2006) found that FMD was not influenced during LBNP, yet only men were examined in these studies and LBNP is conducted in the supine posture without skeletal muscle activation. It is currently unknown if being in the upright posture influences FMD or β2-adrenergic responsiveness in men and/or women. Indeed, Dyson et al. (2010) concurrently found that muscle chemoreflex activation *via* post-exercise circulatory occlusion of the legs did enhance the brachial FMD response, yet they attributed this enhanced dilation to baseline constriction obscuring the hyperemic dilatory response. It is important to note that Dyson et al. (2010) controlled for shear stress by changing the length of time that the forearm was occluded in all trials. During upright posture (sitting and standing), brachial vascular resistance increases in healthy men and women (Edgell et al., 2012), potentially indicating a reduction of shear rate and FMD. A reduction of FMD while upright could contribute to the maintenance of blood pressure while upright due to increased peripheral resistance.

Our lab group recently found that brachial FMD correlated with measures of adenosine or acetylcholine mediated coronary resistance in patients with suspected cardiac microvascular disease (Nardone et al., 2020). At the same time, we found that the natural logarithm of the reactive hyperemia index (LnRHI) as measured by the EndoPAT device correlated with dobutamine mediated coronary resistance (Nardone et al., 2020). Therefore, we are using FMD and LnRHI in the current study as markers of these vasodilatory processes in the supine and upright postures. We hypothesized that (1) in the upright posture, brachial artery shear stress would be lower and therefore FMD responses would be attenuated; (2) due to greater adrenergic responses in the upright posture, LnRHI would be enhanced while upright; and (3) both FMD and LnRHI would be augmented in women compared to men.

## Materials and Methods

### Participants

All procedures were approved by the York University Research Ethics Board and all participants gave written informed consent. We adhered to the Declaration of Helsinki and Title 45, US Code of Federal Results, Part 46, Protection of Human Subjects, and all subsequent revisions and amendments. Two unique cohorts of participants were recruited as the EndoPAT protocols were completed as a follow-up study to the FMD protocol. The FMD protocol consisted of 10 women and nine men, while the EndoPAT protocol included nine women and eight men (Table 1). Participants were excluded if they suffered from any previously diagnosed cardiovascular or pulmonary diseases. Women must have never taken oral contraceptives or have stopped taking them for a period of at least 3 months prior to participating in the study. Women were also excluded if they had been using a hormonal intrauterine device, contraceptive patches, or any other form of hormonal contraceptives. Women were tested in the early follicular phase during days 2–5 of the menstrual cycle. For 12 h before testing, all participants were asked to refrain from: smoking (e.g., cigarettes, vaping, and marijuana), drinking alcohol, drinking caffeine (e.g., coffee and tea), heavy exercise (including sports, resistance training, and moderate to intense aerobic exercises), and eating fatty foods. Height and weight were measured with a standard stadiometer and age, sex, and weekly episodes of moderate to vigorous exercise were by self-report. Estimated VO_2_max was calculated using anthropometrics and the frequency of exercise using the Ainsworth equation (Ainsworth et al., 1993).

**Table 1:** Anthropometrics of women and men who completed FMD or EndoPAT testing.

	FMD trials			EndoPAT trials		
	Women	Men	*p* value	Women	Men	*p* value
*n*	10	8		9	8	
Age (years)	20 ± 1	21 ± 2	0.17	22 ± 3	22 ± 2	0.58
Weight (kg)	73 ± 15	80 ± 21	0.44	63 ± 10	81 ± 17^*^	0.01
Height (m)	1.6 ± 0.1	1.7 ± 0.1^*^	0.04	1.6 ± 0.1	1.8 ± 0.1^*^	0.002
BMI (kg/m^2^)	27 ± 5	27 ± 8	0.95	23 ± 3	25 ± 8	0.21
# of times of exercise/week	1.7 ± 1.3	3.3 ± 1.4^*^	0.03	1.8 ± 1.6	2.3 ± 1.3	0.52
VO_2_ max estimate (ml/kg/min)	36 ± 2	48 ± 6^*^	<0.001	38 ± 3	47 ± 6^*^	<0.001

*Values are means ± SD; BMI, body mass index; and VO_2_, maximum oxygen consumption*.

* *Indicates a sex difference within that trial*.

### Cardiopulmonary Measurements

#### Hemodynamics

Heart rate (HR) was measured using a single lead ECG. Blood pressure (BP) was measured using beat-to-beat finger photoplethysmography (NexFin, BMEYE, Amsterdam, Netherlands), which was calibrated to an automated BP measurement (BPTru Medical Devices, Canada) using the right arm in the supine position. Stroke volume (SV) and subsequently calculated cardiac output (Q) and total peripheral resistance (TPR) were collected using the Modelflow algorithm of Nexfin. SV (and thus Q and TPR) were normalized to body surface area using the Du Bois formula (Du Bois and Du Bois, 1916). All hemodynamic signals were obtained using a Powerlab data acquisition device (1,000 Hz) and LabChart Pro software (ADInstruments, Colorado Springs, United States).

#### Flow-Mediated Dilation

The brachial artery was imaged by author KH approximately 3–5 cm proximal from the antecubital fossa using a linear array high resolution ultrasound transducer (9L-RS; 3–10 MHz) using Duplex ultrasound to concurrently measure blood flow velocity and vessel diameter (Vivid i, GE Healthcare Systems, Mississauga, Canada). Continuous ultrasound images were recorded using a video grabber device (AV.io HD, Epiphan Video) and the blood velocity, brachial artery diameter, and resultant shear rates (baseline and maximal) were analyzed using automated edge-detection software (Cardiovascular Suite, Quipu, Italy). Shear rate (SR) was calculated as SR = 4*(velocity/diameter), where 4 assumes a constant blood viscosity. The FMD protocol consisted of 2 min of baseline measurement, 5 min of forearm occlusion ~50 mmHg over systolic blood pressure, and 3 min of reactive hyperemia. Low-flow mediated constriction was calculated by comparing the brachial diameter at baseline to the brachial diameter during the last 30 s of occlusion.

#### EndoPAT

The EndoPAT device (EndoPAT, Itamar Medical, Israel) was used according to manufacturer’s instructions except that the occlusion cuff was placed on the forearm rather than the upper arm for concurrent measurements of FMD. This technique has been used previously by our group (Nardone et al., 2020, 2021). Briefly, two tonometry finger cuffs were placed on the index finger of each hand and pulse waveforms throughout each cardiac cycle and were relayed to the device and an automated algorithm quantified the reactive hyperemia index (RHI). The EndoPAT protocol consisted of 5 min of baseline measurement, 5 min of forearm occlusion ~50 mmHg over systolic blood pressure, and 5 min of reactive hyperemia. Since the RHI is not normally distributed (Hamburg and Benjamin, 2009), the software computed the natural logarithm of the RHI (Ln-RHI). The EndoPAT 2000 also calculated the augmentation index (AI), typically used as an indirect marker of systemic arterial stiffness, and calculated the AI at a normalized HR of 75 bpm, which serves to allow for comparisons across different populations and postures.

### Experimental Protocol

**Figure 1:**
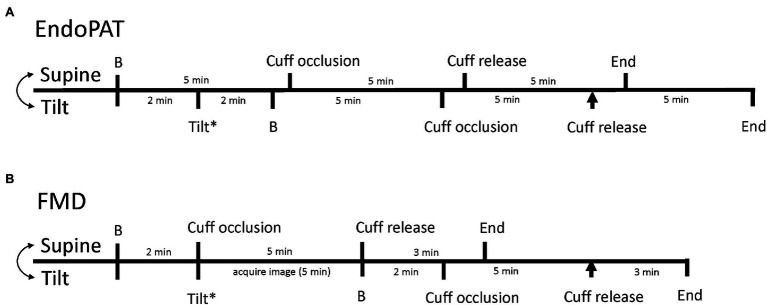
Timeline of data collection for EndoPAT trials **(A)** and flow-mediated dilation (FMD) trials **(B)**. The timelines for the supine trials are on the top of each line and the timeline for the tilted trials are on the bottom of each line. The double headed arrow indicates that the supine and tilted trials are randomized. **B** Indicates baseline, the asterisk highlights the time of upright tilt, and the arrowhead indicates the time of reactive hyperemia/cuff release during tilt.

Each FMD or EndoPAT protocol (one while supine and one while 70° upright) consisted of baseline measurements, forearm occlusion/ischemia, and reperfusion measurements as described above. A standard blood pressure cuff was placed on the forearm of the right arm and for all trials both hands/arms were kept at the level of the heart. For tilted trials, the EndoPAT trial began immediately upon achieving upright posture (~2 min) whereas the FMD trials began within ~5 min due to the necessity of obtaining an adequate ultrasound image in the upright posture. Since the baseline period for the EndoPAT trial is 5 min and the baseline period for the FMD trials is 2 min, we planned for cuff release at approximately the same time between the trials. Supine and upright trials within each methodology were randomized and conducted with a 30-min break between them to minimize any serial effect of the occlusion periods. The protocol is shown in Figure 1.

### Data Analysis

Anthropometric data were compared with an unpaired *t*-test between the sexes. One minute hemodynamic averages were taken at baseline (i.e., 1-min prior to cuff inflation while supine or tilted, as appropriate), and 15 s averages were taken at the time of maximal brachial artery dilation (FMD) or the time of maximal finger blood volume (EndoPAT) after cuff-release/reperfusion. In the tilted posture, the baseline values were taken at least 2 min after onset of tilt. Our lab group previously observed that in healthy young men and women blood pressure stabilized after 2 min of 70° upright tilt (Joshi and Edgell, 2019). Changes in hemodynamics were calculated as the change from baseline to the time of maximum dilation. A two-way mixed model ANOVA was used to compare hemodynamic data across the two postures (sex and posture as factors, posture is a repeated measure). Analysis of vascular variables (i.e., FMD, SR, SR_AUC_, and Ln-RHI) was also done *via* a two-way mixed model ANOVA while accounting for sex and posture (repeated measure) as factors. The normality of distribution was assessed *via* the Spiro-Wilks test of normality. Tukey’s *post hoc* analysis was used when significance was found. Correlations between the change in FMD between postures and the blood pressure responses were conducted with linear regression. Significance was defined as *p* < 0.05. All statistical analyses were performed *via* Sigmaplot 13.2 (San Jose, California, United States) statistical software. Data in tables and text are presented as Mean ± SD. Data in figures are presented as median and the 25 and 75th percentiles.

## Results

For both the FMD and EndoPAT trials, women were smaller than men and had a lower estimated VO_2_max (Table 1; all *p* < 0.05). The baseline brachial artery was smaller in women but did not differ between posture trials (Sex: *p* = 0.002, Posture: *p* = 0.43, and Interaction: *p* = 0.81). At baseline in the tilted posture, both women and men had higher HR, lower mean arterial pressure (MAP), and lower stroke volume index (SVi) compared to the baseline in the supine position (Table 2, all *p* < 0.005). Men had higher baseline cardiac output index (Qi) and SVi compared to women in both supine and tilted trials (Table 2, all *p* < 0.05). There were no effects of sex or posture on total peripheral resistance index (TPRi) or baseline brachial artery shear rate/blood velocity (Table 2, all *p* > 0.05).

**Table 2:** Baseline hemodynamics and brachial artery diameter of women and men prior to starting the FMD protocol in the supine and tilt posture.

	Women		Men		
	Supine	Tilt	Supine	Tilt	Significance
HR (bpm)	69 ± 8	86 ± 8^*^	66 ± 7	84 ± 8^*^	Sex (*p* = 0.42) Posture (*p* < 0.001) Interaction (*p* = 0.83)
MAP (mmHg)	86 ± 9	80 ± 9^*^	85 ± 4	79 ± 8^*^	Sex (*p* = 0.79) Posture (*p* = 0.003) Interaction (*p* = 0.77)
Qi^†^ (L/min/m^2^)	3.4 ± 0.3	3.4 ± 0.3	3.9 ± 0.6	3.7 ± 0.7	Sex (*p* = 0.048) Posture (*p* = 0.53) Interaction (*p* = 0.55)
SVi^†^ (ml)/(m^2^)	45 ± 8	40 ± 5^*^	57 ± 10	47 ± 10^*^	Sex (*p* = 0.013) Posture (*p* = 0.004) Interaction (*p* = 0.45)
TPRi (mmHg/L/min)/(m^2^)	7.4 ± 2.7	7.4 ± 1.9	6.1 ± 1.2	5.9 ± 1.1	Sex (*p* = 0.08) Posture (*p* = 0.77) Interaction (*p* = 0.81)
Brachial artery diameter^†^ (mm)	3.1 ± 0.4	3.3 ± 0.7	3.7 ± 0.2	3.9 ± 0.4	Sex (*p* = 0.002) Posture (*p* = 0.43) Interaction (*p* = 0.81)
Mean brachial blood velocity (cm/s)	13 ± 8	13 ± 11	16 ± 11	14 ± 8	Sex (*p* = 0.67) Posture (*p* = 0.60) Interaction (*p* = 0.65)
Baseline shear rate (/s)	316 ± 181	247 ± 113	216 ± 86	218 ± 63	Sex (*p* = 0.17) Posture (*p* = 0.37) Interaction (*p* = 0.24)

*Values are means ± SD. HR is heart rate, MAP is mean arterial pressure, Qi is cardiac output index, SVi is stroke volume index, and TPRi is total peripheral resistance index*.

† *Indicates significant difference between women and men*.

* *indicates significant difference between tilt and supine posture*.

**Figure 2:**
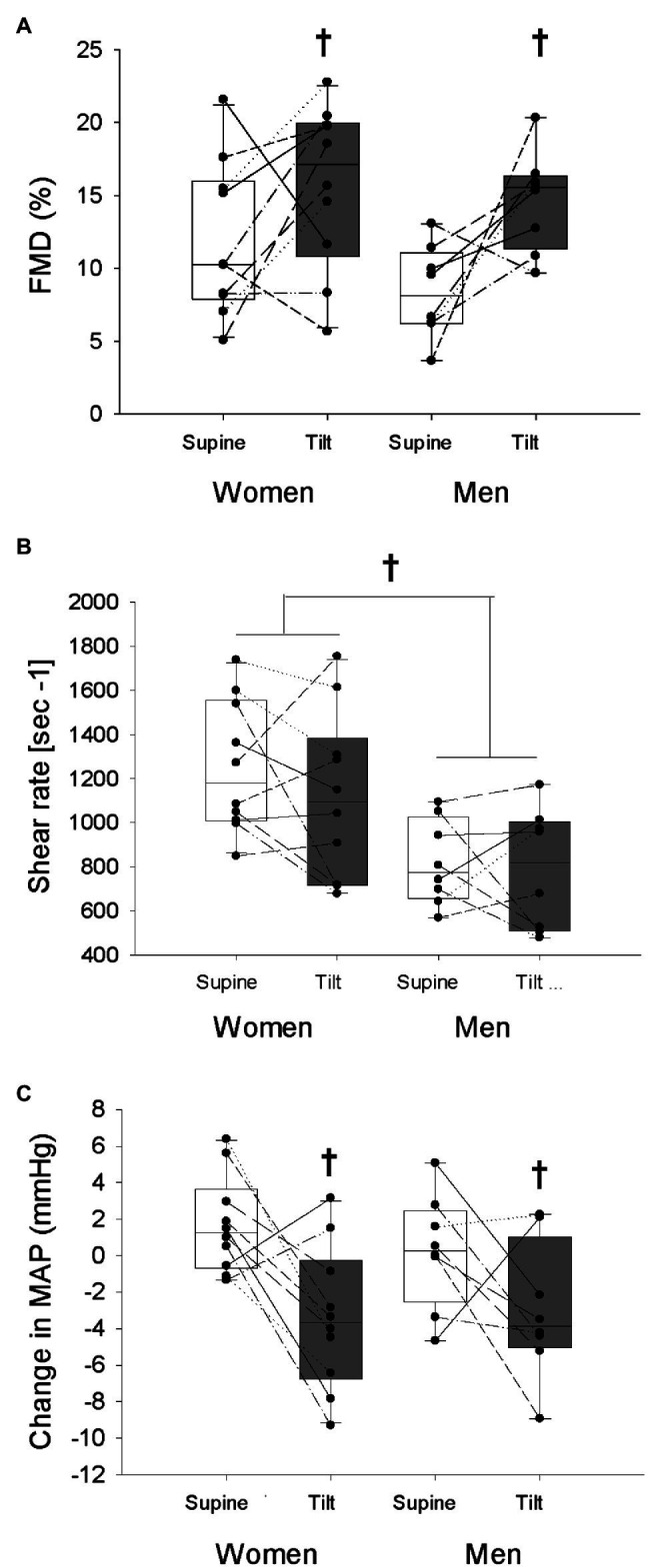
Flow-mediated dilation (FMD; **A**), maximal shear rate **(B)**, and the change in mean arterial pressure (MAP) from the beginning of each trial to the time of maximal brachial artery diameter response **(C)** is shown in women and men in the supine and tilted postures. White bars indicate supine posture, grey bars indicate upright posture. ^†^indicates a main effect of posture (*p* < 0.05). *Indicates a main effect of sex (*p* < 0.05).

The change in brachial artery diameter from baseline to immediately before reperfusion (indicating low-flow mediated constriction) was not different between sexes or postures (Women supine: −0.01 ± 0.14 mm, Women tilt: −0.01 ± 0.34 mm; Men supine: −0.03 ± 0.11 mm, Men tilt: +0.14 ± 0.23 mm; Sex: *p* = 0.20, Posture: *p* = 0.37, and Interaction: *p* = 0.38). Upright tilt enhanced the FMD response in both men and women (Figure 2A; *p* = 0.005) despite no change in shear stress while upright (Figure 2B; *p* = 0.31). Men had lower shear stress during both trials compared to women (Figure 2B; *p* = 0.005). Both sexes had a greater reduction of MAP from the beginning to the end of the FMD trial during the tilted trial compared to the supine trial (Figure 2C; *p* = 0.003). When FMD was normalized to maximal shear rate by division, a significant increase was still observed in both sexes during the upright posture (Women supine: 0.010 ± 0.004 au, Women tilt: 0.015 ± 0.007 au; Men supine: 0.009 ± 0.005 au, Men tilt: 0.019 ± 0.006 au; Sex: *p* = 0.48, Posture: *p* < 0.001, and Interaction: *p* = 0.19). From the beginning to the end of the trials, women had a greater increase of HR regardless of posture (Table 3, *p* = 0.046), and men had a greater reduction of SVi over the course of the trial in the tilted posture compared to the supine posture which was not seen in women (Table 3, Interaction effect *p* = 0.047). There were no effects of sex or tilt on the change in Qi or TPRi over the course of the trials (Table 3, all *p* > 0.05).

**Table 3:** Hemodynamic changes from baseline to the time of maximal dilation of women and men across the supine and tilt in the FMD testing.

	Women		Men		
	Supine	Tilt	Supine	Tilt	Significance
ΔHR (bpm)^†^	1.81 ± 4.08	2.61 ± 4.37	−2.54 ± 4.18	0.91 ± 2.93	Sex (*p* = 0.046) Posture (*p* = 0.11) Interaction (*p* = 0.31)
Δ Qi (L/min/m^2^)	0.08 ± 0.22	−0.02 ± 0.22	0.02 ± 0.31	−0.10 ± 0.22	Sex (*p* = 0.47) Posture (*p* = 0.07) Interaction (*p* = 0.93)
Δ SVi (ml)/(m^2^)	−0.59 ± 5.43	−1.33 ± 1.87	2.46 ± 2.54	−4.40 ± 7.50^*^	Sex (*p* = 1.0) Posture (*p* = 0.02) Interaction (*p* = 0.047)
Δ TPRi (mmHg.min∕L)/(m^2^)	−0.51 ± 1.05	−0.36 ± 0.57	−0.030 ± 0.40	−0.16 ± 0.22	Sex (*p* = 0.22) Posture (*p* = 0.96) Interaction (*p* = 0.42)
Δ Mean brachial blood velocity (cm/s)	40 ± 22	21 ± 15	25 ± 22	17 ± 12	Sex (*p* = 0.14) Posture (*p* = 0.047) Interaction (*p* = 0.40)

*Values are means ± SD. Δ is change from time of baseline to time of maximum dilation during the FMD protocol, Qi is cardiac output index, SVi is stroke volume index, and TPRi is total peripheral resistance index*.

† *Indicates significant difference between women and men in tilt posture*.

* *Indicates significant difference between tilt and supine posture*.

To investigate if the observed fall in MAP over the course of the tilt trial was from the observed increase of FMD, we examined their relationship, yet found none in our combined group of participants [Change in BP = −2.832 − (0.297 × Change in FMD), *R*^2^ = 0.15, *p* = 0.12]. To investigate potential sex differences, the group was separated by sex. The equation for men was [Change in BP = −1.990 − (0.200 × Change in FMD), *R*^2^ = 0.05, *p* = 0.59], and for women was [Change in BP = −3.549 − (0.412 × Change in FMD), *R*^2^ = 0.34, *p* = 0.08].

**Figure 3:**
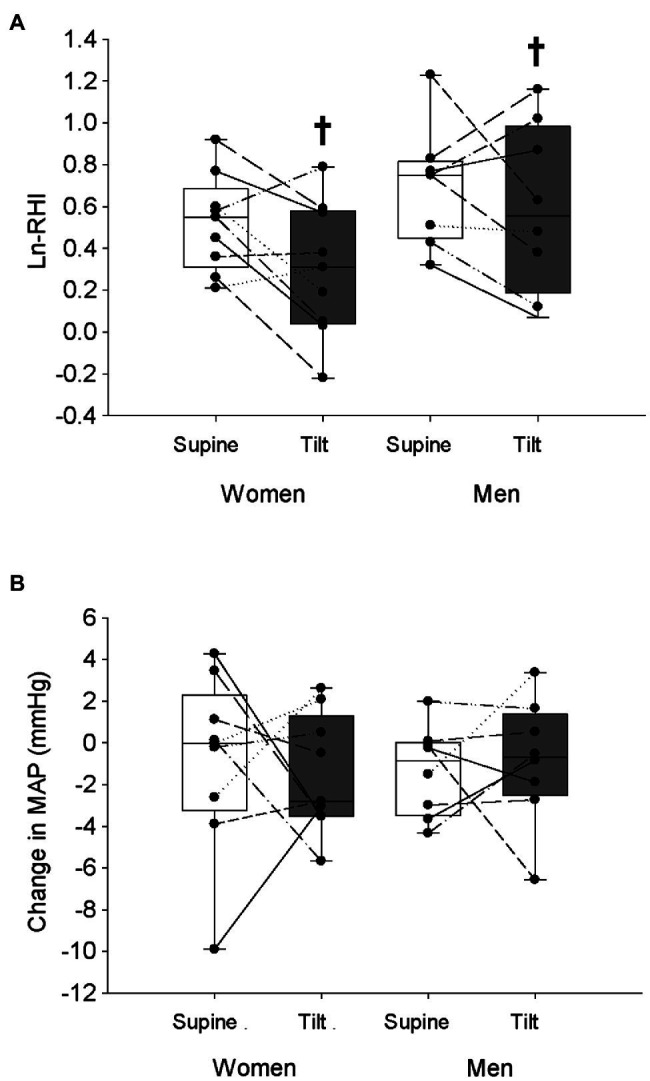
Natural logarithm of the reactive hyperemia index (LnRHI; **A**) and the change in MAP **(B)** from the beginning of each trial to the time of maximal hyperemic response is shown in women and men in the supine and tilted postures. White bars indicate supine posture, grey bars indicate upright posture. ^†^Indicates a main effect of posture ( *p* < 0.05).

For the EndoPAT trials, women had higher TPRi compared to men at baseline (Table 4, *p* < 0.001), both women and men had higher HR and lower SVi at baseline in the tilted trial compared to baseline in the supine trial (Table 4, *p* < 0.001), and there were no effects of sex or posture on baseline MAP, Qi, or TPRi (Table 4, *p* > 0.30). Both men and women displayed a reduction of LnRHI in the tilted posture compared to the supine posture (Figure 3A, *p* = 0.037), yet no posture effect on the change in MAP over the course of the trials (Figure 3B, *p* = 0.78). All participants had a greater increase of HR over the course of the tilted trial compared to the supine trial (Table 5, *p* = 0.049), and there were no effects of sex or posture on the change of Qi, SVi, or TPRi over the course of the trials (Table 5, *p* > 0.05). AI@75 bpm was not different between sexes or with posture change (Women Supine: −10.0 ± 11%, Women Tilt: −8.0 ± 8%, Men Supine: −20 ± 8%, Men Tilt: −16 ± 13%; Sex *p* = 0.070, Posture *p* = 0.13, and Sex × Posture *p* = 0.54).

**Table 4:** Baseline hemodynamics of women and men prior to starting the EndoPAT protocol in the supine and tilt posture.

	Women		Men		
	Supine	Tilt	Supine	Tilt	Significance
HR (bpm)	72 ± 7	85 ± 9^*^	70 ± 10	85 ± 8^*^	Sex ( *p* = 0.78) Posture ( *p* < 0.001) Interaction ( *p* = 0.73)
MAP (mmHg)	84 ± 9	83 ± 10	85 ± 10	84 ± 11	Sex ( *p* = 0.77) Posture ( *p* = 0.78) Interaction ( *p* = 0.93)
Qi (L/min/m^2^)	3.9 ± 0.6	3.7 ± 0.5	4.0 ± 0.8	3.9 ± 0.7	Sex ( *p* = 0.31) Posture ( *p* = 0.67) Interaction ( *p* = 0.74)
SVi (ml)/(m^2^)	55 ± 6	44 ± 5^*^	57 ± 6	46 ± 5^*^	Sex ( *p* = 0.23) Posture ( *p* < 0.001) Interaction ( *p* = 0.98)
TPRi^†^ (mmHg/L/min)/(m^2^)	7.7 ± 1.4	8.5 ± 2.8	5.7 ± 1.2	5.6 ± 0.8	Sex ( *p* < 0.001) Posture ( *p* = 0.62) Interaction ( *p* = 0.47)

*Values are means ± SD. HR is heart rate, MAP is mean arterial pressure, Qi is cardiac output index, SVi is stroke volume index, and TPRi is total peripheral resistance index*.

† *Indicates significant difference between women and men in tilt posture*.

* *Indicates significant difference between tilt and supine posture*.

**Table 5:** Hemodynamic changes from baseline to the time of maximal hyperemia of women and men across the supine and tilt in the EndoPAT testing.

	Women		Men		
	Supine	Tilt	Supine	Tilt	Significance
ΔHR (bpm)	−0.25 ± 3.15	4.91 ± 6.89^*^	0.61 ± 5.55	2.38 ± 5.68^*^	Sex ( *p* = 0.70) Posture ( *p* = 0.049) Interaction ( *p* = 0.31)
Δ Qi (L/min/m^2^)	0.04 ± 0.19	0.11 ± 0.33	0.14 ± 0.31	0.12 ± 0.26	Sex ( *p* = 0.61) Posture ( *p* = 0.81) Interaction ( *p* = 0.66)
Δ SVi (ml)/(m^2^)	0.82 ± 1.69	−1.37 ± 2.15	1.62 ± 2.23	0.26 ± 2.72	Sex ( *p* = 0.07) Posture ( *p* = 0.06) Interaction ( *p* = 0.64)
Δ TPRi (mmHg.min∕L)/(m^2^)	−0.04 ± 0.33	−0.45 ± 0.84	−0.24 ± 0.48	−0.23 ± 0.35	Sex ( *p* = 0.95) Posture ( *p* = 0.31) Interaction ( *p* = 0.29)

*Values are means ± SD. Δ is change from time of baseline to time of maximum dilation during the FMD protocol, Qi is cardiac output index, SVi is stroke volume index, and TPRi is total peripheral resistance index*.

* *Indicates significant difference between tilt and supine posture*.

## Discussion

We found that in men and women vasodilatory responses change in the upright posture equally. Specifically, we observed that the FMD response is enhanced yet the EndoPAT response is attenuated. These changes do not appear to be due to changes in shear stress or hemodynamics.

### Flow-Mediated Dilation

We hypothesized that FMD would be lower in the tilt position due to reduced shear stress; however, we found no change in shear stress and an improved FMD while upright. We also did not observe any sex differences in FMD in either posture. While FMD can be influenced by other contributing factors, FMD is typically proportional to shear stress as its primary mechanism (Pyke et al., 2004). However, in the current study, we observed an increased FMD in the upright posture with no change in shear stress and a reduction in blood pressure. Hence, our results suggest a mechanism other than shear stress causing the increased vasodilation. Further, while we hypothesized that a reduction of FMD while upright would be protective of blood pressure while upright, these results suggest that enhanced FMD while upright could in fact be a contributing factor to reductions of blood pressure while upright.

Guazzi et al. (2005) previously explored brachial FMD while 60° upright in a healthy group of primarily men (10/12 men). However, shear stress was not calculated nor was continuous arterial diameter and blood pressure (measures were taken every 15 s). Nonetheless, our results in both women and men correspond with their findings. Guazzi et al. (2005) also measured brachial diameter in a subset of participants in the contralateral arm during and after hyperemia in tilt and found no changes; however, they combined healthy controls with those with pathophysiologic conditions such as hypertension and type 2 diabetes. Therefore, it remains unknown if the observed enhancement of vasodilation is local or systemic in healthy men and women. Indeed, Dietz et al. (1997) observed that during syncope, sympathetic withdrawal was not sufficient to explain the vasodilation that occurs, and that the vasodilation may act through mechanisms independent of β2-adrenergic and nitric oxide mediated mechanisms.

Rubini et al. (2012) conducted a study investigating the effect of the upright posture on the metabolic, cardiovascular, and electromyographic (EMG) activity of postural muscles (soleus and gastrocnemius) and they observed that the EMG activity of both muscles increased while upright. These results indicate increased muscle activation and presumably increased metabolite production while upright. However, it is important to note that they used a standing model rather than tilt table testing. We suggest that metabolites produced *via* upright posture (and potentially from deltoid and rotator cuff muscles from the elevated arm position of the imaged arm despite support) enter the ischemic forearm upon reperfusion causing an enhanced vasodilatory effect mediated by FMD related mechanisms such as adenosine and potentially lactate. Indeed, enhanced adenosine production is known to lead to syncope (Saadjian et al., 2002) and lactate stays in circulation for approximately 14 min (Almenoff et al., 1989). Hence, it is feasible that metabolites such as lactate stay in the general circulation long enough to enter the ischemic arm upon cuff release and possibly cause the increased non-shear stress dependent vasodilatory effect observed in our results. Systemic plasma concentrations of adenosine and lactate are needed during tilt table testing to determine their role in enhanced vasodilation.

Interestingly Guazzi et al. (2005) found a strong positive association between the change of HR during the tilt trial and the change of brachial artery diameter potentially linking peripheral vasodilation and the tachycardiac response to tilt. However, in the current study, even though MAP decreased and FMD increased during tilt, we did not observe a significant relationship between the changes in blood pressure and FMD. Contrary to our expectations we did not find evidence that women had enhanced FMD compared to men in either posture, yet they did have higher shear stress which is likely due to having smaller brachial arteries. Conflicting evidence about sex differences in the FMD response exist. Johns et al. (2020) suggested these discrepancies could be due to consideration of baseline brachial arterial diameter. For example, Juonala et al. (2008) found that women have greater FMD responses in comparison to men, however, after normalizing for baseline diameter, they found no significant differences in FMD between women and men and after similar normalization Shenouda et al. (2018) found lower FMD in women compared to men. Allometric scaling in the current study would not influence our primary results (i.e., change of FMD in upright posture) as there were no differences in baseline diameter in between postures. We also suggest that measurements of fitness or VO_2_ should be conducted in future studies. In the current study, since the significantly higher shear stimulus in women compared to men did not elicit higher FMD in women, we suggest that our cohort of women have reduced endothelial function compared to our male cohort due to less times exercising per week leading to a lower estimated VO_2_max. VO_2_max has been shown to be correlated with endothelial health (Buscemi et al., 2013).

### EndoPAT

While the EndoPAT has been used to assess overall endothelial function, we used it as an index of assessment for β2-mediated dilation of the microvasculature, as our lab group previously observed that LnRHI is correlated with dobutamine induced vasodilation in the coronary artery (Nardone et al., 2020). We hypothesized that (1) LnRHI would be enhanced in the upright posture because of the increased β2 adrenergic receptor binding from sympathetic activation associated with the upright posture, and (2) in both postures, women would have higher LnRHI compared to men. Neither hypothesis was supported by our findings.

Goswami et al. (2013) investigated LnRHI and posture change in men and women previously and found no effect of sex or posture, however, they measured LnRHI after returning to the supine posture rather than while upright. For the current study, LnRHI was lower in the upright posture in both sexes suggesting reduced β2-mediated dilation in light of enhanced adrenergic responses to tilt. We speculate that prior to cuff release, the β2-receptors were saturated with norepinephrine which entered the ischemic arm *via* sympathetic nerve activity. We suggest that this was not observed as vasodilation prior to reperfusion (i.e., baseline brachial artery diameter did not change in the FMD trials when tilted) due to concurrent vasoconstrictor signals from α1-receptor stimulation. However, upon reperfusion and the associated stimuli, there was a reduced capacity for the β2-receptors to cause further dilation which would have been measured by LnRHI.

We did not observe a sex difference in resting LnRHI which was unexpected as Davis et al. (2020) found that women have greater Ln-RHI scores in comparison to age-matched men. This discrepancy could again be due to the underlying fitness differences of our participants. The men and women in Davis et al. (2020) were matched on the history of regular resistance exercise, and we did not control for fitness or physical activity. Hence, reduced fitness in women for the current study could have been a confounding variable responsible for attenuated vascular function.

### Limitations

Blood pressure fell over the course of the FMD trial, but not throughout the EndoPAT trial. This could have been due to any delays in the length of time that it took to find an adequate brachial artery image upon tilt (despite attempting to control for the time of cuff release). However, despite this fall in blood pressure, shear stress was not affected during reperfusion and FMD increased regardless. For the EndoPAT trials, the relatively higher (i.e., unchanged from baseline) blood pressure compared to the FMD trials could have been expected to increase LnRHI while upright, however, the opposite was observed.

We did not include measurements of muscle metabolism, plasma nitrates/nitrites, and oxidative stress markers, such as peroxynitrite, catecholamines, or other vasoactive substances. In order to test our hypothesis that the increased FMD in the upright posture is due to activation of postural muscles, future studies should consider measuring potentially vasoactive substances, such as lactate, adenosine, O_2_ and CO_2_, nitrates/nitrites, as well as electromyography of postural and shoulder muscles. Along with these measurements of muscle metabolism, we suggest conducting a true cardiopulmonary exercise test to determine fitness, as we used a VO_2_ estimate *via* the Ainsworth equation. Lastly, we suggest measurements of alternate vascular beds (e.g., renal and splanchnic) to determine their role in the maintenance of blood pressure in light of the greater vasodilatory capacity in skeletal muscle beds while upright.

Our calculations of the relationships between the reduction of blood pressure of the course of the tilt FMD trial and the increase of FMD were underpowered. Greater sample numbers would help to strengthen any assumptions, which may be made using those data. Similarly, the statistically significant reduction of LnRHI in the upright posture was underpowered at *β* = 0.48; however, all other statistically significant comparison reached sufficient power of *β* > 0.8.

## Conclusion

We have provided evidence of changes in two distinct vasodilatory pathways that have opposite responses while in the upright posture. FMD increases while LnRHI decreases while upright. These results indicate that while endothelial-dependent vasodilation improves, endothelial-independent vasodilation is impaired. To support these observations, an investigation of the arterial response to sublingual nitroglycerin while upright would be beneficial. Despite the hypothesis that these changes could be partially responses for sex differences in orthostatic tolerance, sex differences were not observed in these cohorts. Larger studies with in-depth investigations of metabolism and fitness are required.

## Data Availability Statement

The raw data supporting the conclusions of this article will be made available by the authors, upon reasonable request.

## Ethics Statement

The studies involving human participants were reviewed and approved by York University Research Ethics Board. The patients/participants provided their written informed consent to participate in this study.

## Author Contributions

KH and HE had substantial contributions to the conception or design of the work. KH, BF, and HE contributed to the acquisition, analysis, or interpretation of data for the work, contributed to drafting the work or revising it critically for important intellectual content, provided approval for publication of the content, and agreed to be accountable for all aspects of the work in ensuring that questions related to the accuracy or integrity of any part of the work are appropriately investigated and resolved. All authors contributed to the article and approved the submitted version.

## Funding

This study was funded by the Natural Sciences and Engineering Research Council of Canada (grant number: 2016-05289).

## Conflict of Interest

The authors declare that the research was conducted in the absence of any commercial or financial relationships that could be construed as a potential conflict of interest.

## Publisher’s Note

All claims expressed in this article are solely those of the authors and do not necessarily represent those of their affiliated organizations, or those of the publisher, the editors and the reviewers. Any product that may be evaluated in this article, or claim that may be made by its manufacturer, is not guaranteed or endorsed by the publisher.
